# Reactive, self-administered malaria treatment against asymptomatic malaria infection: results of a cluster randomized controlled trial in The Gambia

**DOI:** 10.1186/s12936-021-03761-8

**Published:** 2021-06-07

**Authors:** Joseph Okebe, Edgard Dabira, Fatou Jaiteh, Nuredin Mohammed, John Bradley, Ndey-Fatou Drammeh, Amadou Bah, Yoriko Masunaga, Jane Achan, Joan Muela Ribera, Shunmay Yeung, Julie Balen, Koen Peeters Grietens, Umberto D’Alessandro

**Affiliations:** 1 Medical Research Council Unit The Gambia At the London School of Hygiene and Tropical Medicine, Fajara, The Gambia; 2 Department of International Public Health, Liverpool School of Tropical Medicine, Liverpool, UK; 3 Medical Anthropology Unit, Institute of Tropical Medicine, Antwerp, Belgium; 4 Amsterdam Institute of Social Science Research, Amsterdam, The Netherlands; 5 MRC International Statistics and Epidemiology Group, London School of Hygiene and Tropical Medicine, London, UK; 6 Malaria Consortium, Cambridge Heath, London, UK; 7 PASS Suisse, Neuchâtel, Switzerland; 8 Clinical Research Department, London School of Hygiene and Tropical Medicine, London, UK; 9 School of Health and Related Research, The University of Sheffield, Sheffield, UK

**Keywords:** Reactive treatment, Malaria prevalence, Asymptomatic infection, Village health worker

## Abstract

**Background:**

Selectively targeting and treating malaria-infected individuals may further decrease parasite carriage in low-burden settings. Using a trans-disciplinary approach, a reactive treatment strategy to reduce *Plasmodium falciparum* prevalence in participating communities was co-developed and tested.

**Methods:**

This is a 2-arm, open-label, cluster-randomized trial involving villages in Central Gambia during the 2017 and 2018 malaria transmission season. Villages were randomized in a 1:1 ratio using a minimizing algorithm. In the intervention arm, trained village health workers delivered a full course of pre-packed dihydroartemisinin-piperaquine to all residents of compounds where clinical cases were reported while in the control arm, compound residents were screened for infection at the time of the index case reporting. All index cases were treated following national guidelines. The primary endpoint was malaria prevalence, determined by molecular methods, at the end of the intervention period.

**Results:**

The trial was carried out in 50 villages: 34 in 2017 and 16 additional villages in 2018. At the end of the 2018 transmission season, malaria prevalence was 0.8% (16/1924, range 0–4%) and 1.1% (20/1814, range 0–17%) in the intervention and control arms, respectively. The odds of malaria infection were 29% lower in the intervention than in the control arm after adjustment for age (OR 0.71, 95% CI 0.27–1.84, p = 0.48). Adherence to treatment was high, with 98% (964/979) of those treated completing the 3-day treatment.

Over the course of the study, only 37 villages, 20 in the intervention and 17 in the control arm, reported at least one clinical case. The distribution of clinical cases by month in both transmission seasons was similar and the odds of new clinical malaria cases during the trial period did not vary between arms (OR 1.04, 95% CI 0.57–1.91, p = 0.893). All adverse events were classified as mild to moderate and resolved completely.

**Conclusion:**

The systematic and timely administration of an anti-malarial treatment to residents of compounds with confirmed malaria cases did not significantly decrease malaria prevalence and incidence in communities where malaria prevalence was already low. Treatment coverage and adherence was very high. Results were strongly influenced by the lower-than-expected malaria prevalence, and by no clinical cases in villages with asymptomatic malaria-infected individuals.

*Trial registration*: This study is registered with ClinicalTrials.gov, NCT02878200. Registered 25 August 2016. https://clinicaltrials.gov/ct2/show/NCT02878200.

## Background

Where malaria transmission has reduced significantly, it has become extremely heterogeneous, with some locations having higher transmission intensity than surrounding areas. Such locations are characterized by clusters of clinical cases, malaria-infected individuals with little or no symptoms and a variable health-seeking profile in the population [1]. While clinical cases point to on-going transmission, a significant proportion of malaria-infected individuals are asymptomatic, with low parasite densities over long periods, and able to infect mosquitoes [2]. Interventions that target these clusters with the aim of clearing the human reservoir of infection could accelerate the transition to elimination in areas on the cusp of reaching this milestone [3].

The concept of mass drug administration (MDA) and other versions of mass treatment campaigns have received renewed interest in the context of malaria elimination. These interventions involve repeatedly treating large swathes of the population with a full course of an anti-malarial, without ascertaining individual infection status. This assumes that, if implemented for a sufficiently long period, treatment would clear the human reservoir of infection and hence interrupt transmission [4].

MDAs are used against many infectious diseases [5] and were an essential strategy in some countries that achieved malaria elimination [6]. However, such levels of success have been difficult to replicate in recent clinical studies and treatment campaigns [7, 8]. The inability to reach and sustain the required levels of coverage is one of the main constraints to producing the anticipated impact on transmission. These treatment campaigns are expensive and difficult to implement efficiently. Currently, MDA is considered only for areas on the verge of interrupting transmission, with good access to treatment, effective vector control and surveillance systems, and minimal risk of re-introduction of infection [4].

There are other variants of mass drug treatment aimed at reducing infections in specific at-risk populations such as pregnant women and young children [9, 10]. These have been very successful and provide complementary evidence that targeted approaches can reduce the burden of disease in high-risk populations [11].

With improvements in malaria case management and surveillance, populations at high risk of infection and disease have been detected using data on passively reported malaria cases [12]. There is also evidence that clusters of asymptomatic parasite carriage and clinical cases can be spatially related; hence close contacts of a malaria patient are more likely to be infected compared to a random sample of the population [13]. Based on this premise, treating these “household contacts” for infection could further reduce transmission [14].

This “reactive” treatment has been implemented in some countries in sub-Saharan Africa [15, 16] and Asia [17]. The overall impact has been positive though, as with MDAs, it is influenced by several factors such as the local epidemiology [18], delivery strategy, response time [19], efficiency of local health systems [20] and underlying community structures [21]. There is clearly the need for evidence on reactive malaria treatment to support countries considering malaria elimination [22].

The Gambia has reported a significant reduction in the malaria burden over the last 15 years [23, 24] and is one of the few sub-Saharan African countries where interrupting transmission may be feasible [25]. To support this process, a trans-disciplinary approach was used to co-develop a reactive treatment strategy with participating communities, to deliver a full course of dihydroartemisinin-piperaquine (DP) to individuals living in the same cluster of houses (compound contacts) of confirmed malaria patients attending the local health services. The efficacy of the intervention was tested in a cluster-randomized trial. The hypothesis was that timely reactive treatment of compound contacts of malaria cases, delivered through family members, can reduce both the incidence of clinical malaria during the transmission season and the prevalence of infection at the end of the transmission season.

## Objective

The objective of this study was to determine whether prompt and systematic self-administered malaria treatment by residents in a compound where a clinical malaria case occurred, reduces *Plasmodium falciparum* prevalence at the end of the transmission season.

## Methods

### Trial design

**Fig. 1:**
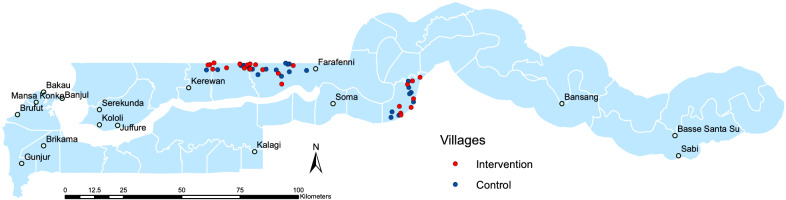
Map of the study area

This was a two-arm, open-label, cluster-randomized controlled trial conducted in the Central Gambia region (Fig. 1). The unit of randomization was the village with trial procedures delivered to all residents in a compound based on the allocation of the village. The trial was conducted between June and December, the period of malaria transmission in The Gambia, in the years 2016–2018. The intervention was delivered between August and December each year, with post-intervention surveys conducted between November and December, in 2017 and 2018. At the start of each study season, sensitization campaigns and census surveys were carried out to create a record of compound residents in the study area.

As part of the site preparation, messages about the trial objectives were curated by social scientists in the research team based on the views of the study population on malaria burden, access to care during illness, delivery of treatment and self-administration of medicines in the household [26]. These messages were refreshed to the communities during the sensitization campaigns.

### Trial location and study participants

Malaria transmission in The Gambia is seasonal (July–December) and varies in intensity across the country, with incidence of clinical malaria during the transmission season varying between 1.7 episodes/person-years in eastern Gambia to 0.2 and 0.1 episodes/person-years in central and western Gambia, respectively [27]. Malaria diagnosis and treatment (artemether-lumefantrine is the first-line treatment) services are available for free in all government clinics; in large communities (> 400 residents), trained resident village health workers (VHW) can perform diagnosis by malaria rapid diagnostic test (RDT) and treat positive cases for the residents of their community.

Villages on the North Bank East and Lower River health regions were purposely identified based on available data on malaria prevalence from field surveys [28] and clinical records of health facilities within the study area. Malaria prevalence by molecular methods in 2012 was 4.59% in the North Bank region and 9.36% in the Lower River region [28]. Each village is structured around family units or compounds. Each compound is defined by a location where one or several households made up of members of an extended patrilineal family reside. The compounds are usually headed by the oldest man in the family and his roles range from resource management to making health-related decisions. The living areas in the compound are organized into living areas for the household head, married women who share them with their children, and older boys.

Individual written informed consent for trial activities, including enumeration and cross-sectional surveys (all villages), was collected from residents of selected study villages. Additional consent was collected for procedures specific to the trial arm. In the intervention arm, consent to measure individual body weight and to deliver treatment if a malaria case was identified in the compound was collected. In the control arm, consent was obtained to screen residents of compounds where a malaria case was reported. Consent procedures were updated for new members of the compounds identified during the pre-season sensitization or when study procedures were to be conducted in the compound. The information provided was used to develop a logbook listing of residents by compound, which was used by the VHW to deliver treatment or by the research team to screen compound members. The details are presented in the trial protocol [29].

VHWs are an integral part of the local health system [30]. To embed the trial within the local health system, the VHWs were trained on how to deliver pre-packed doses of DP to compound residents of a malaria case, how to elicit and report on drug-related adverse events after treatment and to retrieve empty or unused treatment from compound residents. The smaller study villages that did not have VHWs were invited to identify a village resident who would report on confirmed cases and on adverse events to the study team. Such ‘village collaborator’ was trained to deliver questions on adherence to treatment and retrieve empty or unused treatment packs. Treatment for compounds without a resident VHW was delivered by a research nurse.

### Randomization

Villages were randomized to one of the two arms using a computer-generated minimization algorithm by the trial statistician. Concealment of allocation was not feasible as all participants and study field staff were aware of village allocations, nor blinding of the treatment was feasible. However, laboratory staff processing samples were blinded to the source of the samples as these were labelled with unique codes delinked from the study identifiers.

### The intervention

**Fig. 2:**
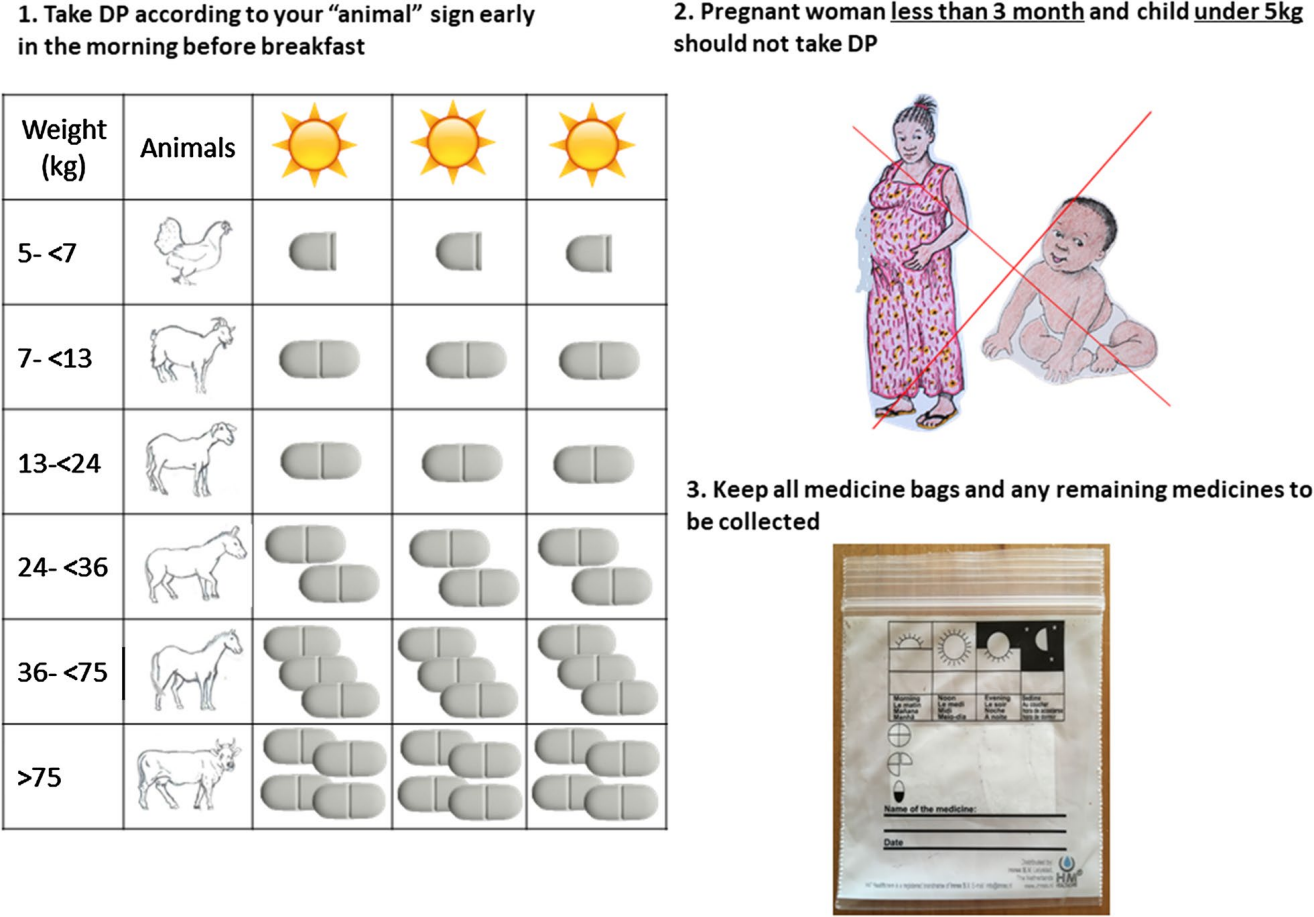
Poster message on use of dihydroartemisinin-piperaquine handed to “treated” compounds

The intervention comprised treating with a full treatment of weight-calculated dose of DP all residents of a compound where a malaria case was reported. To ensure ease and prompt delivery, VHWs were given a start-up pack that included a treatment box containing pre-packed doses of DP, a logbook with residents listed by compound, and weight-derived, pictorial treatment chart developed during the trial preparation sessions with the community leaders (Fig. 2). The pre-packed doses were prepared based on the manufacturer’s weight recommendation and labelled with stickers corresponding to the pictures on the logbook. The drugs were replenished by the research nurse after each compound had been treated.

Residents in all study villages were encouraged to visit their VHW when they were unwell for a malaria test. When an individual tested positive for malaria, they were treated with artemether-lumefantrine according to national guidelines. The VHW then visited the compound of this index case and delivered to the compound head, with instructions on how and when it should be taken, sufficient DP doses to treat all residents. VHWs visited the compound 4 days later to retrieve unused medicines and empty medicine packs, and record any adverse events.

A study nurse accompanied the VHW during their first visits to compounds to be treated. Subsequent visits were carried out by the VHW alone. In case of a new resident or person without a written informed consent, the VHW informed the supervising study nurse who visited the compound and obtained the informed consent before administering treatment.

### Procedures in the control arm

In the control arm, VHWs treated index cases with artemether-lumefantrine and reported these events to the study nurse who visited the compound within 5 days to collect a finger-prick blood sample from all residents. These samples were transferred to the central laboratory where they were stored until molecular diagnosis for *P. falciparum* infection. Residents who were symptomatic at the time of the visit were offered an RDT and, if positive, treated with artemether-lumefantrine.

### Sample processing

*Plasmodium falciparum* DNA was isolated from dried blood spots using an automated QIAcube HT extractor robot (Qiagen^®^). Positive and negative controls were included during extraction to assess efficiency of DNA extraction and the risk of contamination, respectively. 5 µL of extracted DNA was used in an ultrasensitive real-time PCR assay [31] with 3D7 standards of known parasite concentration serially diluted and used to generate standard curves and set limits of parasite DNA detection. Quantification cycle (Cq) values of test samples above the limit of detection scored as positive. Samples with borderline Cq values were scored independently by a second reader and, where results remained discrepant, DNA from the sample was re-extracted and the process repeated. All results were analysed using the BIO-RAD CFX96 Touch™ Real-Time PCR detection system.

### Endpoints

The primary endpoint was *P. falciparum* prevalence, by molecular methods, in all age groups at the end of the intervention period. Following disruptions due to local political unrest in the country and a very short rainy season in 2017, the trial was extended for 1 year with the trial endpoint deferred to the end of the 2018 transmission season.

For the cross-sectional surveys, the sample size needed to show a 60% difference in prevalence between the arms was estimated assuming a 5% prevalence in the control arm, 80% power and 5% significance level. A random sample of all listed residents was drawn from each village, in proportion to the village size. Each village was informed through the resident VHW about the survey and once specific dates were agreed, the VHW disseminated the information to the village. On the scheduled day, study teams with support from volunteers, approached the selected persons in the compounds and invited them to meet at a central location where blood sampling was done. Selected persons who were absent or refused to participate were not replaced on the list. A finger-prick blood sample was collected onto Whatman filter paper, air-dried, and transported to the laboratory for *P. falciparum* parasite detection using the same protocol described above [31].

The secondary endpoints included the incidence of clinical malaria cases (diagnosed by RDT) as detected through the health system, in both intervention and control clusters, and treatment coverage as determined by percentage of individuals living in the same compound having received and taken at least 80% of the prescribed dose. For this, a combination of the count of medicine packs and tablets returned and the VHW feedback where medicine packs were not accounted for was used.

### Sample size consideration

It was assumed the intervention would decrease malaria prevalence by at least 60%, i.e., from 5 to 2% or less [28]. With 34 villages, 17 per arm, the trial would be able to detect such effect at 80% power, 5% significance level and 0.7 coefficient of variation. However, in November 2017, malaria prevalence in the control arm was lower than expected (2.8%). After approval by the Steering Trial Committee and the funder, 16 additional villages in the South Bank of the river Gambia, 8 per arm were added to increase the power of the study.

### Data management and statistical analysis

Information on treatment delivered, adherence and retrieval of blister packs and unused medicines were recorded on paper forms by the study nurse at scheduled visits to each VHW. The study nurses also collected information on persons screened for infection in control villages. These forms were submitted to the field station where they were checked for consistency by the study coordinator before double-entry by data clerks. Forms were entered onto the trial database on OpenClinica (OpenClinica, LLC). Laboratory results were directly exported onto a Microsoft Access database. Data from the field were linked with the laboratory results during analysis.

The data analysis followed an agreed statistical analytical plan based on the published protocol [29]. The prevalence of malaria infection was calculated as the proportion of sampled population during the end-of-season surveys with a positive PCR result; the period prevalence of clinical episodes was defined as the number of positive malaria cases detected by RDT, in the entire study population during the intervention period (August–December). Both outcomes were compared between arms using a random effects logistic regression model to give an odds ratio of the effect of the intervention.

### Changes to protocol

Two unexpected events during the trial informed additional data analyses and are presented as “unplanned analysis”. The extension of the trial for an additional year/transmission season provided an opportunity to assess the effect of the intervention across two transmission seasons. Unexpectedly, many villages in both arms and in both years did not record any clinical case and thus, if in the intervention arms, did not receive any treatment. For this reason, the results are presented as per a pre-specified analysis plan and an unplanned analysis, restricted to villages where at least one clinical case was reported. Since this unplanned analysis included fewer clusters, the data over the two study years were combined to maintain statistical power. In the cross-sectional survey results, an unplanned subgroup analysis by age is reported.

## Results

**Fig. 3:**
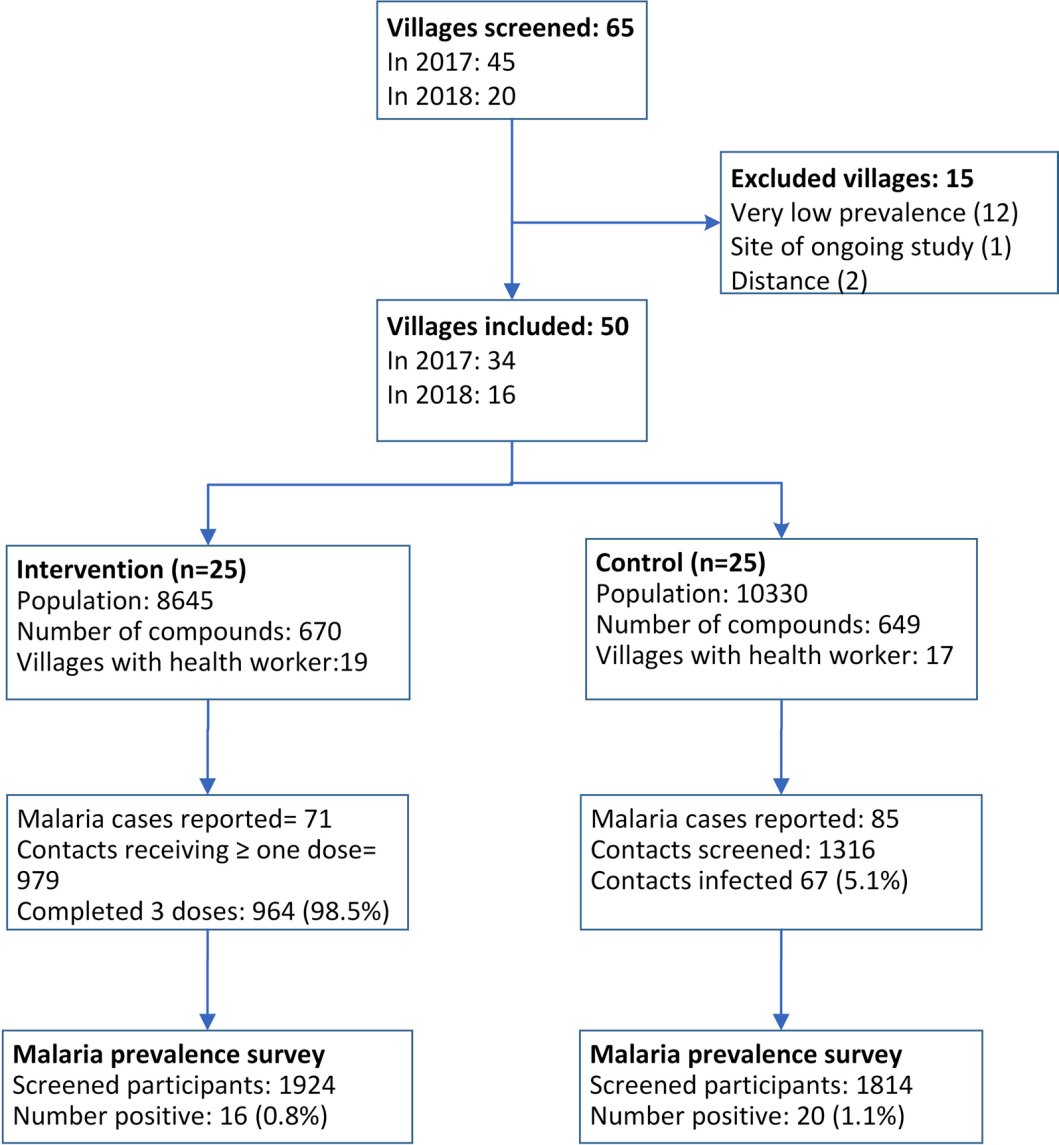
Consort flow diagram for the trial

Sixty-five villages were purposely screened for eligibility: 45 and 20 in the 2017 and 2018 malaria transmission seasons, respectively. Of this, 50 were included in the trial; 34 in the 2017 season and 16 additional villages in the 2018 season. Reasons for exclusion are shown in the trial flow diagram (Fig. 3).

The study population comprised 18,975 residents in 1319 compounds, with a median of 269 (range 34–2147) residents per village. The number of compounds were similar in both arms (Table 1). About half (51.7%, 9816/18,975) of the population and 45.8% (604/1319) of compounds were in the 34 villages in the North Bank. About half (51.6%) of the population was female, with no significant difference in gender distribution between arms. In the population who knew their age (82.5%, 15,657/18,975), the median age was 15.5 (range 0.1–87.9) years, with 12.7% (1982/15657) below 5 years. Thirty-six (72.0%) villages had a resident VHW, 19 of them in villages in the intervention arm.

**Table 1:** Baseline characteristics of study area and population

	Total	Intervention	Control
Number of villages	50	25 (50.0%)	25 (25.0%)
Villages on the North bank	34	17 (68.0%)	17 (68.0%)
Number of compounds	1319	670 (50.8%)	649 (49.2%)
Population	18,975	8645 (45.6%)	10,330 (54.4%)
Female	9790	4486 (45.8%)	5304 (54.2%)
Villages with resident village health worker	36	19 (52.8%)	17 (47.2%)
Median age (range) in years*	15.6 (0.04–97.58), n = 15,681	15.23 (0.04–97.56), n = 6325	15.86 (0.05–99.98), n = 9356
Population by age group*			
Under 5	1957	852 (43.5%)	1105 (56.5%)
5–14	5665	2271 (40.1%)	3394 (59.9%)
15–30	3703	1468 (39.6%)	2235 (60.4%)
Over 30	4356	1734 (39.8%)	2622 (60.2%)

^*^Based on 82.6% (15,681/18,975) of the population that provided their age

### Planned analysis

#### End-of-season malaria prevalence

In 2018, malaria prevalence at the end of the transmission season was 0.8% (16/1924, range 0–4%) and 1.1% (20/1814, range 0–17%) in the intervention and control arms, respectively (Table 2). After adjusting for age, prevalence was not significantly lower in the intervention compared to the control arm (OR 0.71, 95% CI 0.27–1.84, p = 0.48).

**Table 2:** The prevalence of parasite infection and period prevalence of clinical cases for all study villages in 2018 (n = 50)

Outcome	Intervention	Control	Odds ratio^a^ (95% confidence interval)
Infection prevalence			
All clusters^b^	0.8% (16/1924)	1.1% (20/1814)	0.71 (0.27, 1.84) p = 0.48
North bank	0.3% (4/1246)	0.1% (1/1134)	3.58 (0.4, 32.1) p = 0.255
South bank^a^	1.8% (12/665)	2.8% (19/669)	0.61 (0.29, 1.26) p = 0.182
By age			
Under 5 years	0.4% (1/237)	3.5 (7/199)	0.11 (0.01, 0.94), p = 0.043
5–14 years	0.5% (4/783)	0.7% (5/769)	0.84 (0.22, 3.17), p = 0.8
15–30 years	1.9% (6/323)	0.7% (2/279)	2.76 (0.55, 13.9), p = 0.219
Above 30 years	0.9% (5/567)	1.1% (6/555)	0.88 (0.26, 2.91), p = 0.83
Period prevalence of clinical malaria			
All clusters	0.8% (71/8645)	0.8% (85/10330)	1.04 (0.57, 1.91) p = 0.893
North bank	0.2% (6/3752)	0.2% (13/6064)	0.77 (0.23, 2.54) p = 0.664
South bank	1.3% (65/4893)	1.7% (72/4266)	0.81 (0.34, 1.92) p = 0.613

^a^Random effects logistic regression models are not valid with a small number of clusters per arm so a *t* test on cluster level summaries was used; in these cases, a risk ratio is presented instead of an odds ratio

^b^Adjusted for age

In 2017, malaria prevalence was 1.8% (23/1259) and 2.4% (39/1638) in the intervention and control arms, respectively. The prevalence was not significantly lower in the intervention compared to the control arm after adjusting for age (0.54, 95% CI 0.21–1.37, p = 0.193, Table 3).

**Table 3:** The prevalence of parasite infection and period prevalence of clinical cases for all study villages in 2017 (n = 34)

Outcome	Intervention	Control	Odds ratio (95% confidence interval)
Infection prevalence^a^	1.8% (23/1259)	2.4% (39/1638)	0.54 (0.21, 1.37), p = 0.193
By age			
Under 5 years	1.1% (2/191)	3.7% (10/2720)	024 (0.05, 1.31), p = 0.101
5–14 years	1.7% (9/538)	1.6% (11/698)	0.91 (0.29, 2.81), p = 0.865
15–30 years	3.0% (6/201)	3.6% (9/252)	0.64 (0.18, 2.31), p = 0.498
Above 30 years	1.8% (6/333)	2.1% (0/425)	0.81 (0.23, 2.8), p = 0.734
Period prevalence of clinical malaria	0.3% (12/3752)	0.3% (18/6064)	0.95 (0.30–2.97), p = 0.930

^a^Random effects logistic regression models are not valid with a small number of clusters per arm so a *t* test on cluster level summaries was used; in these cases, a risk ratio is presented instead of an odds ratio

#### Period prevalence of clinical malaria cases

**Fig. 4:**
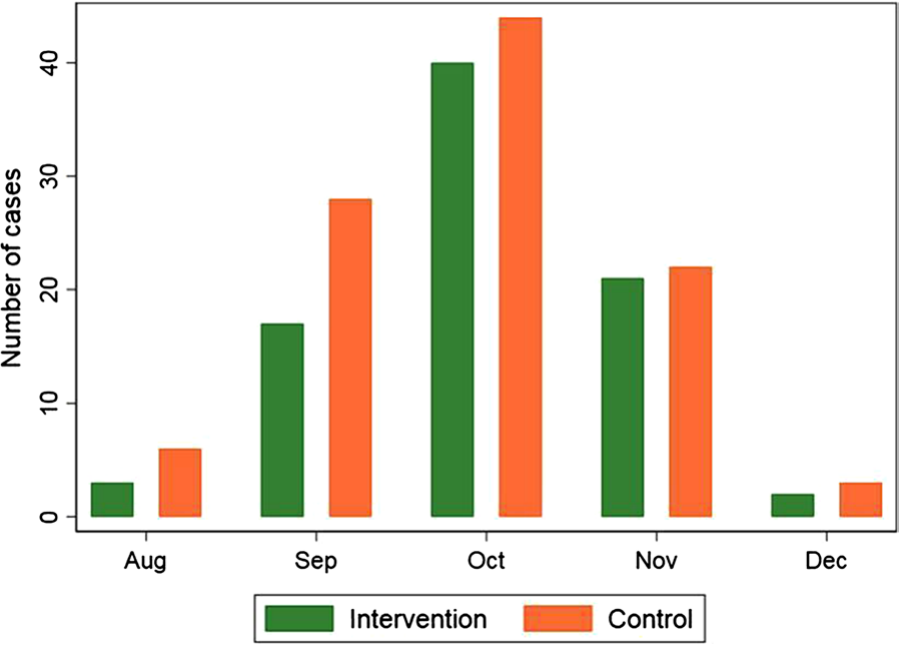
Distribution of clinical cases in the transmission season (both seasons combined)

The peak in clinical cases occurred between September and November (Fig. 4). During the 2018 season, 71 cases were reported in 11 of the 25 intervention villages: eight villages in the South Bank and three villages in the North Bank (Table 2). Only six (8.5%, 6/71) of the reported cases were from the North Bank, each of them from different compounds. There were 85 clinical cases reported in 14 (14/25, 56.0%) control villages, 13 (15.3% 13/85) of the cases were from the North Bank. There was no difference in period prevalence of clinical malaria between the intervention (0.8%, 71/8645) and the control (0.8%, 85/10330) arm (OR 1.04, 95% CI 0.57–1.91).

During the 2017 season, there were 12 malaria cases reported in five (29.4%) of the 17 intervention villages, with 239 registered residents in these compounds, with the median compound size of 17 (range 5–37) residents. In the control arm, 18 clinical cases were reported from seven villages.

#### Treatment coverage and adherence (intervention arm only)

Compound visits were carried out for all recorded cases. In 2018, there were 1014 residents listed in the compounds of index cases, with the median of 12 (range 3–60) residents per compound. Most listed residents (96.6%, 979/1014) were present at the time of the visit and received treatment. Adherence to treatment was high as 98.5% (964/979) of those treated completed the full 3-day treatment. In 2017, most of the compound residents (95.0%, 227/239) were treated and, among these, 96.5% (219/227) completed the three doses of treatment.

#### Parasite infection among screened compound residents (control arm only)

The period prevalence of infection in screened compounds, i.e., at the time a case of clinical malaria was diagnosed, was 5.1% (67/1316); the population prevalence in the control arm, measured at the end of the transmission season, was 1.1% (20/1814). When comparing period prevalence with the population prevalence at the end of the transmission season, malaria prevalence was on average 2.2% (95% CI 0.3%–4.2%, p = 0.027) higher in compounds with a clinical case.

In 2017, the prevalence among screened compound residents was 3.3% (33/1014) while population prevalence at the end of the transmission season was 2.9% (49/1679). When comparing these two estimates, malaria prevalence was on average 0.6% (95% CI −7.5%, 8.8%, p = 0.853) higher in compounds with a clinical case.

### Unplanned analyses

#### Malaria prevalence in 2017 and 2018 seasons

When combining 2017 and 2018, the average malaria prevalence was 1.2% (39/3183, range 0–10.5%) in the intervention arm and 1.7% (59/3452, range 0–13.3%) in the control arm (OR 0.65, 95% CI 0.37–1.15, p = 0.138).

#### Malaria prevalence in villages reporting at least one malaria case

Over the course of the study, 37 villages, 20 in the intervention and 17 in the control arm, reported at least one clinical case (Table 4). When combining both years and adjusting for age, prevalence was about 50% lower in the intervention than in the control arm (OR 0.51, 95% CI 0.3–0.86, p = 0.013). The difference in malaria prevalence between study arms was particularly marked in children under 5 years of age (OR 0.12, 95% CI 0.2–0.58, p = 0.009).

**Table 4:** End-of-season prevalence of infection in study villages where at least one clinical case was reported

Outcome	Intervention	Control	Odds ratio (95% confidence interval)
Infection prevalence			
2018 (all clusters)	0.9% (14/1629)	1.4% (20/1399)	0.51 (0.18, 1.41), p = 0.194
North bank	0.2% (2/952)	0.1% (1/726)	1.64 (0.14, 18.1), p = 0.688
South bank	1.8% (12/677)	2.8% (19/673)	0.61 (0.29, 1.26), p = 0.182
2017	1.2% (12/975)	2.4% (32/1322)	0.51 (0.25, 1.05) p = 0.07
Combined both seasons	1.0% (26/2604)	1.9% (52/2721)	0.51 (0.3, 0.86) p = 0.013
By age			
Under 5 years	0.5% (2/368)	3.9% (15/382)	0.12 (0.02, 0.58), p = 0.009
5–14 years	0.8% (8/1072)	1.2% (14/1140)	0.59 (0.21, 1.64), p = 0.311
15–30 years	1.9% (8/428)	2.5% (10/403)	0.71 (0.24, 2.1), p = 0.532
Above 30 years	1.1% (8/736)	1.6% (13/795)	0.61 (0.22, 1.75), p = 0.361

## Adverse events reported during drug treatment

In 2018, 32 (84.2%) of the 38 adverse events were considered as mild and 6 as moderate. In 2017, 37 adverse events were recorded, all were categorized as mild. No severe adverse event was reported in both years. The moderate adverse events were headache (reported twice), diarrhoea, abdominal pain, joint pain, and cough (Table 5).

**Table 5:** Adverse events, by year, in participants who received dihydroartemisinin-piperaquine

Adverse event	2017	2018	Total n (%)
Vomiting	8	3	11 (14.7)
Loose stools	4	6	10 (13.3)
Diarrhoea	0	7	7 (9.3)
Dizziness	3	4	7 (9.3)
Nausea	6	1	7 (9.3)
Body aches	5	1	6 (8.0)
Abdominal pain	1	4	5 (6.7)
Headache	2	3	5 (6.7)
Tiredness	2	3	5 (6.7)
Weakness	4	1	5 (6.7)
Others	2	5	7 (9.0)
Total	37	38	75 (100.0)

## Discussion

Systematic and timely administration of an antimalarial treatment to all residents of compounds with clinical malaria patients may reduce malaria prevalence. However, prevalence at the end of the 2018 transmission season was not significantly different between study arms, partly due to the lower-than-expected prevalence and to the presence of asymptomatic malaria-infected individuals in villages without a single case of clinical malaria throughout the whole transmission season. Indeed, the recorded prevalence of 1.1% in the control arm at the end of the study was greatly different from the initial assumption of 5%. Almost half of the intervention villages, mostly in the North Bank, did not report any clinical malaria case and thus did not receive any treatment. Nevertheless, clustering of asymptomatic infections around clinical cases seems to occur; in control villages, malaria prevalence in compounds with clinical cases was higher than the population-level prevalence recorded at the end of the transmission season survey. This suggests some asymptomatic malaria-infected individuals do not easily transmit their infection to the vector, particularly when parasite/gametocyte densities are low [32]. Most infections detected at the end of the transmission season surveys were of low density and not associated with symptoms. The low malaria prevalence in these villages, often below 5%, and the low probability of transmission from the few infected individuals to the vector would explain the lower-than-expected effect of the intervention [32]. Nevertheless, malaria prevalence in children under 5 years of age was substantially lower in the intervention than in the control villages, and consistently so in both study years although it reached statistical significance only in 2018 (Table 2), suggesting the intervention was efficacious in this age group.

Trials of reactive treatment in other low transmission settings have also showed similar effectiveness without reaching statistical significance [33], highlighting the influence of background fluctuations in malaria transmission on the impact of interventions designed to interrupt transmission.

The malaria burden in the study area was low [34, 35] and transmission may have been extremely localized, with clinical episodes representing spikes of transmission within compounds but not sufficiently intense to spread to the whole village. The only vector control measures implemented in the region are insecticide-treated nets (ITN), whose last distribution was done during the study, in 2017. In the study area, ITN coverage rates was high, above 70% [27], making this area suitable for the evaluation of interventions such as reactive treatment targeting residual transmission. Reactive treatment relies on the identification of clinical cases to target treatment to individuals likely to be infected. However, there may be clusters of malaria-infected individuals without necessarily the occurrence of clinical cases [33], thus diluting the effect of the intervention.

This approach for delivering the intervention considered social structures in the community; the compound as a microcosm of the community, the VHW’s role as the health expert and local social groups, providing a link between research and the formal health system. By involving these stakeholders (including VHWs, village development groups, traditional birth attendants) in the development and the adaptation of the intervention, e.g., malaria case reporting, drug delivery and administration, high treatment coverage and adherence in both seasons was achieved [26], 19,21. In addition, a protocol of regular contact between research nurses and VHWs was rigorously applied to review malaria reports and discuss trial progress during monthly meetings. Together with the community engagement, it is unlikely clinical cases were missed.

Community members were aware of the direct and indirect impact of malaria. They considered that the treatment given to compound residents was beneficial as it protected vulnerable individuals within the family while resulting in economic benefits from time spent away from work during the farming season if sick, and savings on cost of medicines [26]. This underscores the importance of trans-disciplinary studies aimed at aligning community and programme expectations when planning mass treatment interventions [30].

While not recommended, an unplanned analysis was carried out to better understand the effect of the intervention and the influencing factors. Including in the analysis only villages with at least one clinical malaria case, both for the intervention and control arm, is justified by the fact that villages without any clinical case did not receive any intervention. With hindsight, a different approach, although logistically challenging, could have been to set up a malaria surveillance system over a larger area and recruit throughout the transmission season only villages with clinical cases as soon as these were reported.

When including only villages with at least one clinical malaria case and after adjusting for age, prevalence in the intervention arm was about 50% lower than in the control arm, both in 2017 and 2018, although the trial remains underpowered for the initial estimated effect. These results are consistent with the assumption of localized transmission and infection progressing to clinical disease in non-immune members of the population. Indeed, the lower risk of infection in children under 5 years suggests that these were recent infections successfully treated by the intervention. This range of effect is reported from similar studies [33, 36]. However, none of these studies were able to show statistically significant differences following the intervention. A pooled analysis of these studies would be useful to better understand the impact and role of reactive interventions in interrupting malaria transmission. It would also feed into modelling studies to determine settings where the impact is maximized in reducing or interrupting transmission. Studies to test the effectiveness of long-term markers of transmission [37, 38] and monitoring variability in the risk of transmission in such low transmission settings [39, 40] are needed to assess the long-term impact of reactive treatment on parasite carriage and malaria transmission.

Mass treatment campaigns are characterized by synchronous treatment of defined populations within a fixed period and effectiveness is linked to levels of coverage and adherence to treatment. Studies on reactive treatment have applied a “campaign approach” and report on operational challenges with hard-to-reach places, response times and ensuring coverage for at risk persons [16, 19, 41]. By integrating health messages and using resources close to the “target treatment area”, it was possible to deliver treatment timely, with consistent high coverage and adherence rates. This highlights the value of investing in methods for delivering interventions, acknowledging that “one-size-fits-all” would not be able to achieve optimal coverage. In addition, scalability of the delivery approach should consider how to adapt the research-specific procedures to context. These include using existing enumeration data that is updated when a compound needs to be treated and using age or height derived estimates for dosing.

A key challenge to the process was ensuring the VHW had sufficient quantities of RDTs and artemether-lumefantrine throughout the transmission season. Due to reporting requirements for these commodities, illiterate VHWs could not receive RDTs even if they had been trained for their use. This was addressed by engaging with the regional health team and exploring ways to better support VHWs. These included allowing a literate member of the VHW’s family to complete the report and requisition forms for RDT and artemisinin-based combination therapy (ACT), and deliver supplies when visiting the VHW for supervision.

## Conclusion

Reactive, community-based targeted interventions delivered through local health structures attended by clinical cases may reduce malaria prevalence at the end of the transmission season. However, the trial was not sufficiently powered to show a significant effect due to a lower-than-expected malaria prevalence in the study area and the presence of asymptomatic malaria-infected individuals in villages without any clinical malaria case. Both treatment coverage and adherence levels were high, with DP well tolerated and without major safety concerns.

## Data Availability

The datasets generated in the current study are available from the corresponding author on reasonable request.

## Abbreviations

MDA: Mass drug administration
DP: Dihydroartemisinin-piperaquine
VHW: Village health worker
RDT: Malaria rapid diagnostic test
PCR: Polymerase chain reaction
Cq: Quantification cycle
DNA: Deoxyribonucleic acid
varATS: Var gene acidic terminal sequence
DSMB: Data and safety monitoring board
ACT: Artemisinin-based combination therapy
